# Mayday - integrative analytics for expression data

**DOI:** 10.1186/1471-2105-11-121

**Published:** 2010-03-09

**Authors:** Florian Battke, Stephan Symons, Kay Nieselt

**Affiliations:** 1 Center for Bioinformatics Tübingen, University of Tübingen, Sand 14, 72076 Tübingen, Germany

## Abstract

**Background:**

DNA Microarrays have become the standard method for large scale analyses of gene expression and epigenomics. The increasing complexity and inherent noisiness of the generated data makes visual data exploration ever more important. Fast deployment of new methods as well as a combination of predefined, easy to apply methods with programmer's access to the data are important requirements for any analysis framework. Mayday is an open source platform with emphasis on visual data exploration and analysis. Many built-in methods for clustering, machine learning and classification are provided for dissecting complex datasets. Plugins can easily be written to extend Mayday's functionality in a large number of ways. As Java program, Mayday is platform-independent and can be used as Java WebStart application without any installation. Mayday can import data from several file formats, database connectivity is included for efficient data organization. Numerous interactive visualization tools, including box plots, profile plots, principal component plots and a heatmap are available, can be enhanced with metadata and exported as publication quality vector files.

**Results:**

We have rewritten large parts of Mayday's core to make it more efficient and ready for future developments. Among the large number of new plugins are an automated processing framework, dynamic filtering, new and efficient clustering methods, a machine learning module and database connectivity. Extensive manual data analysis can be done using an inbuilt R terminal and an integrated SQL querying interface. Our visualization framework has become more powerful, new plot types have been added and existing plots improved.

**Conclusions:**

We present a major extension of Mayday, a very versatile open-source framework for efficient micro array data analysis designed for biologists and bioinformaticians. Most everyday tasks are already covered. The large number of available plugins as well as the extension possibilities using compiled plugins and ad-hoc scripting allow for the rapid adaption of Mayday also to very specialized data exploration. Mayday is available at http://microarray-analysis.org.

## Background

Since their inception in the early 1990s, DNA microarrays have revolutionized many areas of biological research. They are a fast and relatively inexpensive tool used for genome-wide studies of gene expression, epigenetic modifications, binding sites of DNA-binding proteins, copy-number variation as well as for resequencing projects. Their success is largely due to the ever growing number of features that can be represented on a single array, allowing for the simultaneous investigation of a large number of genomic loci.

Yet the large number of features, and a concomitant increase in the number of experiments conducted (such as fine-grained time-series experiments), also poses the problem of finding the data of interest. Essential to any microarray experiment is thus the filtering of the large data matrix, the aim is to find (full-width) submatrices ("clusters") with common characteristics. Furthermore, assigning statistical significance values to the features (row-vectors of the matrix) is a very common task. A large number of different methods have been developed for automated as well as exploration-driven analysis of complex data, some of them specific to the field of microarray analyses, others are more general in application.

However, most of these methods are available only as stand-alone programs or proof-of-concept implementations. During a normal microarray experiment, several of these methods have to be used in combination. Which methods are used and in what order depends on the nature of the data, the experimental conditions and on observations made *during the analysis *itself. Thus, bioinformaticians need an integrative framework combining many of these methods to be able to efficiently analyze their data. Such a framework must also allow the quick addition of new methods and support their development via rapid prototyping.

BRB-ArrayTools is such an integrated software system developed by biostatisticians [1]. It is an add-in to Microsoft Excel under the Microsoft Windows family of operating systems. Among the tools are algorithms for normalization, the computation of differentially expressed genes, cluster analysis, and class prediction. BRB-ArrayTools focuses mainly on the development of new statistical methods for expression data analysis.

EMMA 2 provides a wide collection of algorithms and a database to store, retrieve, and analyze genome-wide datasets in a MIAME and MAGE-ML compliant format [2]. For the user it features a web interface, however no offline version is available. EMMA's main emphasis is the analysis of MAGE-compliant data. It is fully open-source offering a large number of various analysis algorithms encompassing preprocessing and normalization, statistical methods for the detection of differentially regulated genes, various cluster algorithms and visualization features. The user can setup pipelines that allow automatic analysis.

The Gene Expression Profile Analysis Suite (GEPAS) offers a similar approach to the analysis of microarray data as EMMA [3]. It also provides a web-based interface. Its main strength is the multitude of tools offered ranging from preprocessing to functional profiling.

The TM4 suite of tools consist of four major applications, Microarray Data Manager (MADAM), Spotfinder, Microarray Data Analysis System (MI-DAS), and Multiexperiment Viewer (MeV), as well as a Minimal Information About a Microarray Experiment (MIAME)-compliant MySQL database [4]. MeV is a microarray data analysis tool written in Java. It is free, open-source software incorporating algorithms for clustering, visualization, classification, statistical analysis and biological theme discovery. MeV offers a number of visualizations. However, it does not allow users to interactively explore data through the combined use of several different linked plots and does not offer many possibilities for using meta information to enhance visualizations.

The importance of appropriate visualization methods for microarray data has long been recognized. A framework for the visual integration of additional meta-information of gene expression data was introduced in [5] and demonstrated in an application of the heat colormap. The enhanced heatmap showed the clear advantages of the integration of supplemental data from different sources for the visual exploration of microarray data.

As the raw experimental data is the biologists' most valuable resource, researchers want to be able to perform their analyses in-house, preferably on their personal computer. The size of modern datasets also makes repeated transfers over the network infeasible.

### Mayday

Mayday [6] is a platform-independent framework for data analysis and visualization. Written in Java, it can be installed locally or run without any installation as WebStart application. Mayday provides efficient core data structures as well as a powerful plugin management system which allows for fast extension via custom plugins. A large number of plugins is already available, covering such areas as clustering, classification, and visualization. All methods presented here are implemented in Java except for the import from Affymetrix CEL files (see below).

Clustering is one of the most common tasks in microarray analyses. Mayday offers several clustering methods with different optimization criteria. Besides the well-established partitioning methods such as *k*-means and SOM [7,8], hierarchical clustering methods such as UPGMA, WPGMA and Neighbor-Joining are available. All clustering methods can be performed with a wide range of distance measures (among them Euclidean, Minkowski, Pearson correlation distance, many more).

Offered visualization tools should be of great assistance in interpreting the results of microarray experiments. Among the most commonly used ones are heatmaps, boxplots, MA scatter plots and histograms. Thus, Mayday's main strength lies in visualization and visualization-driven data exploration. Data can be visualized in many different ways, including profile (parallel coordinate) plots, box plots, scatter plots and heatmaps. All Mayday plots can be exported as publication quality files, using different bitmap formats (JPG, PNG, TIFF) as well as the scalable vector graphics format (SVG). The different views on the data are linked so that interaction with a profile plot is reflected in a simultaneously opened heatmap, for instance. Meta-information can be used to *enhance *the plots, i.e. add additional data to the visualizations. These can come from clusterings (cluster ids) or external sources (e.g. Gene Ontology identifiers), or can be the result of statistical tests applied within Mayday, such as *p*-values. These can, for instance, be used to add additional columns to Mayday's heatmap, to sort the heatmap's rows, to add transparency or a second color dimension or to change the height of rows according to their significance. Furthermore, users can inspect all meta information associated with the probes in a tabular view, sort the table by any meta information column, or use meta information to filter probes.

Finding significantly differentially expressed genes is one of the core functions offered by Mayday. A host of different methods are already available (e.g. Student's *t *test, SAM [9], etc.) and can be combined with correction methods for multiple testing. ANOVA analyses are supported as well.

## Implementation

**Figure 1:**
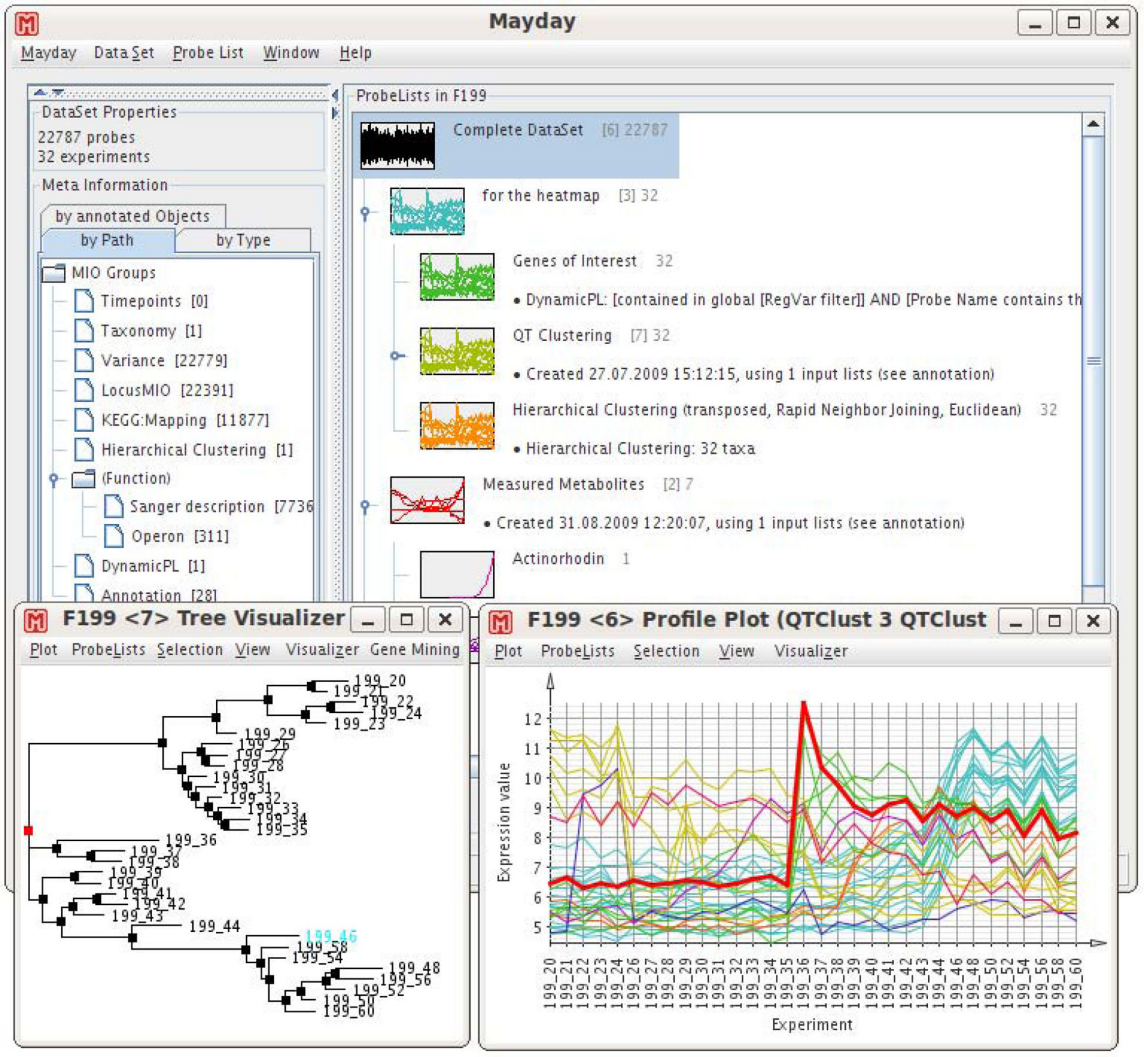
**Main window screenshot**. Mayday's main window during the analyses of the *Streptomyces coelicolor *dataset. Meta-information is shown hierarchically on the left, the hierarchy of probe lists with their preview images on the right. Insets show interactive plots of hierarchical column-wise clustering (left) as well as profile plots of selected clusters (right).

The current version of Mayday offers many enhancements and new features. The core structures were optimized and rewritten to improve performance and simplify the addition of new functionality. Among the new features are the ability to create a hierarchical structure within datasets, a much-improved user-interface with customizable profile previews, matrix operations such as merge and split, new statistical methods for the identification of differentially expressed genes (WAD [10], Rank Product [11]), online data transformations (e.g. *z*-scoring, smoothing, centering) and many more. See figure 1 for an overview of Mayday's user interface. Some of the highlights will be presented in the following sections.

### Automated Processing

Since many analysis steps are common to the first-level analysis of virtually all microarray data, Mayday offers a powerful processing pipeline construction framework allowing for the automation of such tasks and their rapid application to new data sets. Pipelines can be stored persistently and shared with other users.

### Dynamic Filtering

A dynamic filtering framework has been integrated into Mayday, to create so-called Dynamic Pro-beLists. By chaining together any number of filter-ing modules and logical operators, arbitrarily complex filters can be created in an easy to use graphical editor. A large number of modules are available for filtering on expression values, meta data, feature names, the content of other (dynamic) ProbeLists or similarity measures (query-by-example). Dynamic ProbeLists react to changes in the underlying data and are updated accordingly.

### New clustering methods and visualizations

While *k*-means is one of the most used clustering algorithms in microarray analyses, new methods have been developed that overcome some of *k*-means deficits and have been shown to give good results. One such method is quality-threshold (QT) clustering [12], now available in Mayday. Instead of a predefined number of clusters, the input parameter is the desired quality (the radius) of clusters to be found. We have implemented a graphical interface that aids users in determining the correct parameter values for their dataset, depending on the distance measure of choice. Furthermore, a density-based clustering [13] method has been added. Clustering result quality can now be assessed using silhouette plots and different clustering methods can be compared with each other or with a partitioning defined by *a priori *knowledge.

To speed up hierarchical clustering of large datasets, we included an efficient implementation the rapid neighbor-joining algorithm [14]. The trees produced by all hierarchical clustering methods are now stored and can be attached to heatmap plots in addition to being displayed in separate viewers using different layout algorithms.

We extended the idea of Sequence Logos [15] to visualize the general direction of expression within experiments: The ProfileLogo plot shows stacked probe expression bins, scaled to their frequency within each experiment. Expression bins are defined by thresholds, e.g. for up and down-regulated genes. Histogram plots have been implemented to gain insight into the distributions of statistical and experimental values, as well as meta data values attached to the data.

Selected probes resp. genes in each plot can be used as the basis for database queries in a large number of public databases, among them NCBI, Ensembl, Gene Ontology, KEGG, and PubMed.

### Machine Learning

Training, evaluation and application of classification models of numerous different types are further applications of Mayday. For dimensionality reduction and identification of marker genes several feature selection methods are available. The machine learning techniques are provided using the WEKA [16] library which has been integrated into Mayday. In addition, the Gene Mining plugin provides a number of methods to select genes separating classes among the experiments.

### Project management

Mayday's ProjectDB implements central and organized storage of datasets and can be used for data mining purposes. As back-end it can either use Apache Derby [17] (included in the Java WebStart version) or dedicated database management systems (PostgreSQL, MySQL). Datasets can be organized in Projects and Project States, allowing to take snapshots of different stages of their analysis. The graphical ProjectDB browser provides previews of each object, including profile plots and boxplots of the experimental data. The data can also be queried directly using an interactive shell.

Alternatively, Mayday implements a snapshot file format that can be used to save the current state of a data set including meta-information, de-fined clusters, hierarchical clustering trees etc. The snapshot format is specifically designed for fast data storage and retrieval while still being a very space-efficient compressed representation of the data.

### Programmers' access

**Figure 2:**
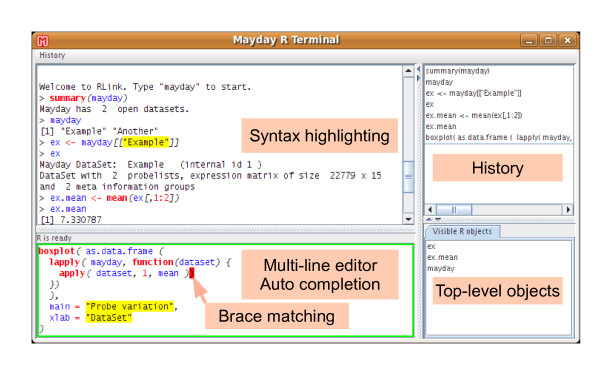
**R Terminal**. Mayday's R terminal offers syntax highlighting, a multi-line editor with context-based auto-completion, a command history and an interactive list of user objects in the global R environment.

Bio*informaticians *will especially like our programmers' access to the data. We have a tightly integrated efficient R shell that integrates the full functionality of R [18] and its wealth of available packages and thus allows the application of third-party methods directly on Mayday's data. R processes can also be connected to Mayday over the network allowing complex calculations to run on a powerful workstation or cluster and communicating with a Mayday instance running on the researcher's laptop, for instance. Furthermore, all gene expression data and meta information currently opened in Mayday can be queried using standard SQL, including the possibility to create new views and custom tables. These shells both feature syntax-highlighting editors with persistent history, greatly increasing programmers' productivity (see figure 2).

### Cross-dataset analyses

Time series analyses as well as replicate studies often require researchers to compare different datasets, e.g. to find systematic shifts in expression over time.

Mayday now offers a specialized view for this purpose in addition to the cross-dataset analyses possible with our R and SQL command-line interfaces.

### Integrated analyses - Systems biology

For integrative pathway analyses, biochemical pathways from several sources, including KEGG [19] and MetaCyc [20] can be visualized as networks. The expression data of enzymes and concentration data of metabolites can be summarized and visualized on the network in different forms, including profile plots and heatmaps.

Gene annotations can be imported from external databases. We currently offer direct support for the Gene Ontology [21] and KEGG databases. Gene identifier mapping can be done automatically using the PICR [22] service.

## Results

### Application study: Dynamic architecture of the metabolic switch in S. coelicolor

To demonstrate the new functionalities of Mayday, we present here an analysis of a large time series in *Streptomyces coelicolor*. For streptomycetes it has proved very difficult to identify the key regulators that control expression of the pathway specific regulators. Mayday was used to monitor the expression dynamics of the bacterium in a time series dataset with unprecedented resolution.

A custom-designed Affymetrix array containing 22,779 probe sets interrogating genes, intergenic regions, and predicted noncoding RNAs was used to study the gene expression in mostly hourly intervals starting at 20 h after inoculation, up to 60 h [23]. Altogether, 32 time points were studied. Phosphate was depleted in the medium at 36 h.

All oligos of the probe sets were mapped to their genomic locus on the chromosome or on one of the two plasmids of *Streptomyces coelicolor*. For each probe set the start and end genomic coordinate together with the strand orientation were written to a tab-separated file.

Within Mayday we imported data from 32 CEL files using Mayday's R interpreter. For normalization we used the robust multi-array average method (RMA) [24] as provided in the affy[25] package of BioConductor [26]. We imported genomic locus information from the tab-separated file described above for later steps in the analysis.

Using a custom processing pipeline, we automatically compute regularized variance for each probe and then apply a filtering step to create a probe list of most variant probesets. Of 22,779 probesets, 64 remain after filtering with a regularized variance threshold of 0.3.

**Figure 3:**
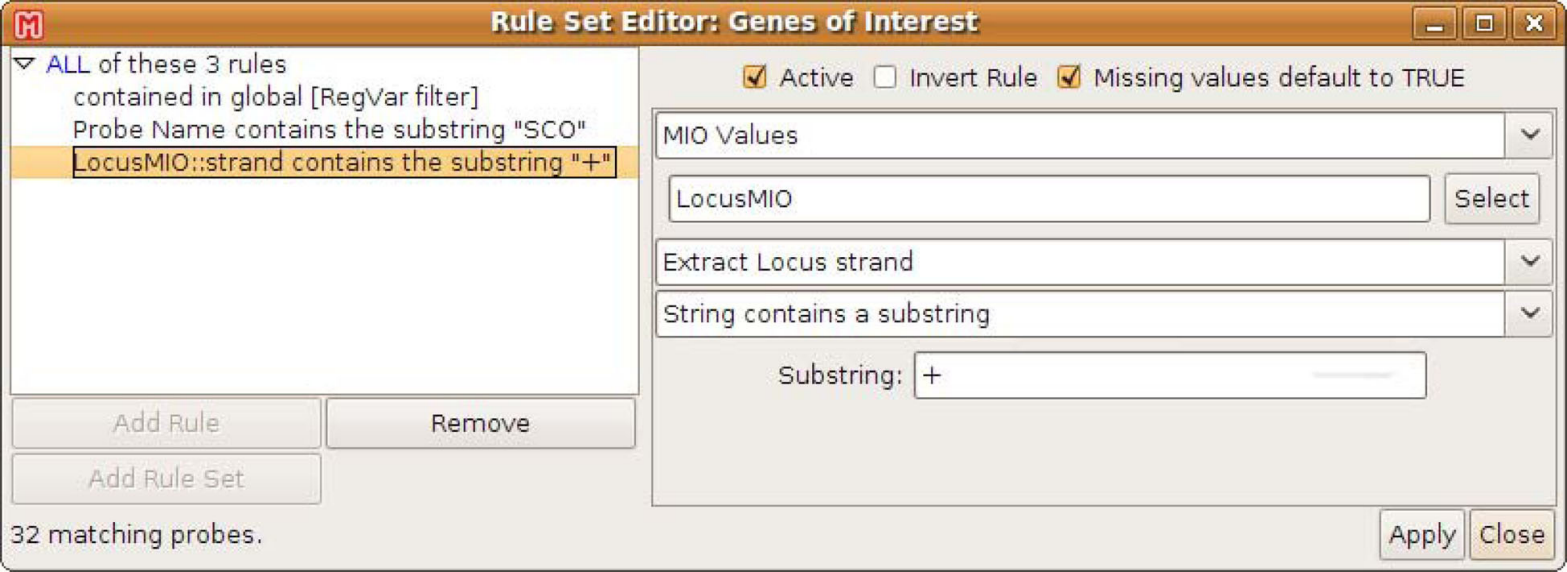
**Rule editor for a dynamic probelist**. Single rules can be arranged via drag & drop into a hierarchy of groups (a rule set) using boolean operations (AND, OR). The number of probes matching the rule set is indicated in the bottom-left corner. Descriptions are automatically created from each rule's content.

Based on this probelist of variant probesets, we create a new dynamic probelist to select only those probes that, apart from being the most variant, interrogate protein coding genes (SCOxxxx), and query the plus strand of the *Sco *genome (see figure 3). 32 probesets remain. Changing any of the filter parameters automatically updates all plots based on the dynamic probelist.

**Figure 4:**
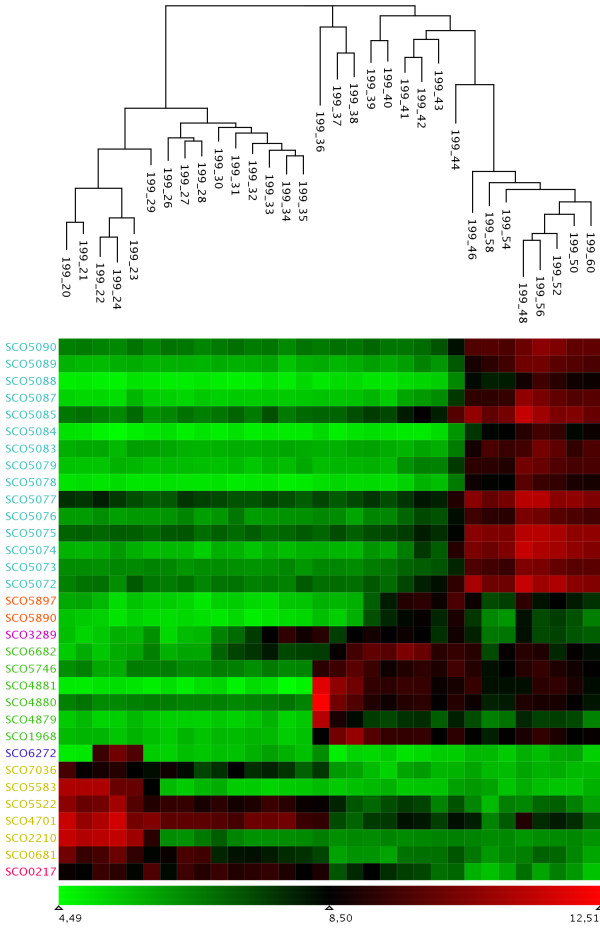
**Heatmap of the clustered experiments**. The heat map shows expression values mapped to a color gradient from low (green) to high expression (red). Experiments are arranged according to a hierarchical clustering dendrogram. The order of genes and the color of gene identifiers is determined by the QT clustering (for details refer to the text) which is also used in figure 5.

The time series sampling reflects the development of *Streptomyces coelicolor *from early growth phase to stationary phase. Accordingly, the expression differences between the samples taken at two consecutive time points should, in general, be smaller than those between samples from time points that lie further apart. Furthermore, the differences between time points should reflect the rate of change in the metabolic state of the culture. To assess this hypothesis, we performed a hierarchical clustering of the transposed matrix, i.e. clustering of the experiments, using the most variant genes. We used the Euclidean distance and MAYDAY's implementation of the rapid neighbour-joining algorithm [14]. The resulting cluster tree is visualized along with a heatmap in figure 4. As expected, the early (20 h) and late time (60 h) points are at the outermost leaves of the tree and consecutive time points are clustered very closely together. The tree nicely depicts the consecutive points of time along the growth curve of the organism. It also shows the major expression change occurring between 35 and 36 hours after inoculation. This largest expression change coincides exactly with the time of complete phosphate depletion in the fermenter.

**Figure 5:**
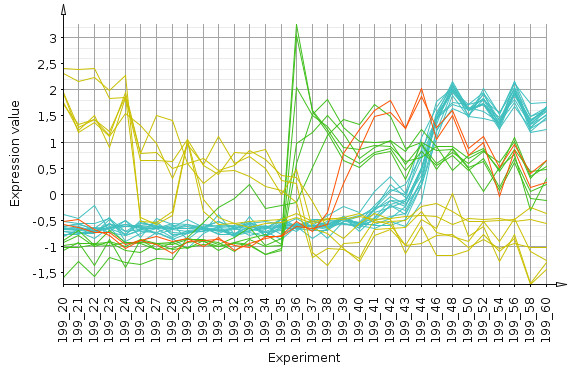
**Profile plot after QT clustering**. The profile colors are determined by the QT clustering (for details refer to the text). Values have been *z*-scored for presentation clarity.

Since the heatmap suggests the existence of distinct groups of genes within the probelist, we use QT clustering with a diameter of 0.4 and use the resulting clusters to color a profile plot showing the *z*-scored profiles of the genes (figure 5). The dynamic architecture of the metabolic switch is clearly visible with different groups of genes being up-resp. down-regulated in a successive order of time points (35, 39 and 43 hours in this subset).

The heatmap also shows that there are some genes that clearly separate the time points 46-60 from the earlier ones. Using the GeneMining plugin, we search for those genes that optimally separate these two groups of experiments (using the quartet mining algorithm, for details see MAYDAY's website). Of the 32 genes in the dynamic probelist described above, 15 belong to the list selected by the quartet mining algorithm. These genes all exclusively belong to the actinorhodin pathway, a genomic cluster of genes (SCO5071-SCO5092).

**Figure 6:**
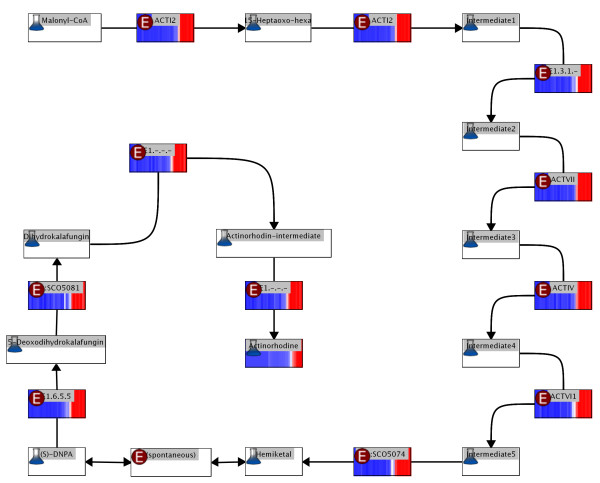
**Actinorhodin pathway**. The actinorhodin pathway (based on ScoCyc pathway information) is visualized with overlaid enzyme expression values and and spectrometrically measured metabolite concentration colored using a red-blue gradient (red = low, blue = high). Consecutive time points are laid out from left to right in the gradient with the leftmost vertical line representing the first experiment (20 h after inoculation) and the right-most vertical line representing the final measurement (at 60 h after inoculation). Enzymes/reactions are marked with the letter "E", metabolites are marked with the image of an Erlenmeyer flask, both are labelled with the identifiers assigned by ScoCyc.

The experimental data also contains optical measurements of the amount of actinorhodin produced. Combining ScoCyc [27] pathway information, expression values and external measurements of actinorhodin levels, we produce an interactive visualization of the actinorhodin pathway (figure 6). On first glance, it is obvious that spectrometrically measured actinorhodin concentration rises in response to the upregulation of several enzymes in this pathway. Interesting target compounds for analysis can be selected from the pathway image for further wet-lab investigation.

**Figure 7:**
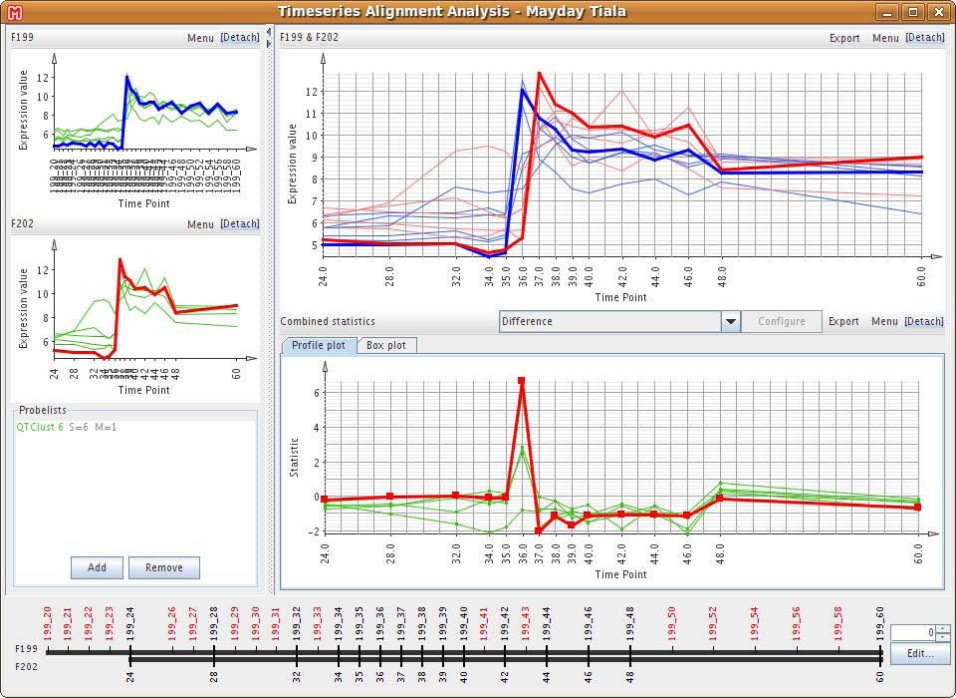
**Mayday's Time series Analysis Window**. The alignment of two time series experiments (F199, F202) shows a time shift by one hour. Our time series alignment tool shows the original datasets (left column), corresponding profiles of a gene in both datasets (top-right) as well as user-defined statistics computed from the corresponding profiles (here the fold-change is used, bottom-right).

Since the dataset used here is part of a larger experiment where biological replicates were produced in separate fermentation runs, we decided to investigate whether we could detect systematic differences between these replicates. Figure 7 shows Mayday's time series alignment tool with one of the QT clusters as an example. The genes in that cluster are up-regulated one hour later in the second fermentation (F202) than in the reference fermentation (F199). This time shift could be traced to a one-hour delay in phosphate depletion in the second fermentation.

## Discussion

Mayday is a comprehensive platform for the analysis and the visual exploration of microarray data. According to Allison et al. [28] the most important statistical components of a microarray experiment analysis involve the following steps: design, preprocessing, inference or classification and validation. During the last years analysis of microarray data has become highly sophisticated, new methods are published almost daily. These range from preprocessing and normalization to novel statistical and machine learning methods. A software that wants to keep pace with these developments has to provide possibilities to enable the rapid integration of new methods as well as making them as usable as possible.

An important focus of exploration of high-dimensional data, such as microarray data, lies on visualization. The advantage of our design is the tight integration of both analysis and visualization as well as the various visualization techniques themselves.

This combination of automatic and visual analysis leads to a visual analytics approach that provides more insights in the structure of the data. We think that with Mayday such a visual analytics approach for the analysis of high-dimensional microarray data has been realized.

## Conclusions

We present a very versatile open-source framework for efficient microarray data analysis, designed for biologists and bioinformaticians. All common tasks of microarray analyses are already covered and the wide range of functionality from the already existing plugins can swiftly be extended with new plugins written in Java, ad-hoc scripting interfaces facilitate rapid prototyping of new algorithms as well as interactive specialized data exploration. Mayday's interactive visualization methods in conjunction with the meta-data concept provide significant insight into complex data and have successfully been applied in many microarray analyses.

New methods and tools are continuously added to Mayday's platform to keep up with new developments. Our coming release includes two new visualizations based on genomic locus information: A track based visualization and a view showing expression (or meta information) values as colored boxes aligned to a linear chromosome laid out continuously in stacked rows. Both are fully interactive and integrated with all other visualizations.

Most recently, novel ultra-high throughput DNA sequencing technologies have been developed that enable researchers to obtain the complete genomes of organisms faster and at a lower cost than classical methods [29]. Moreover, these technologies can be applied to measure gene expression (RNA-Seq) [30] and protein-DNA interactions (ChIP-Seq) [31], and many current studies use RNA-Seq and microarray data comparatively. Our new genomic plots will be especially useful in the context of such new types of data. We're currently working on an integration of these new data types into Mayday, separately or in multi-platform settings.

## Availability and requirements

• **Project name**: Mayday

• **Project home page**: http://microarray-analysis.org

• **Operating systems**: Platform independent

• **Programming languages**: Java

• **Other requirements**: Java 6 or higher

• **License**: GNU GPL version 2

## Authors' contributions

SY and FB are coordinating development of Mayday. FB performed the data analysis and wrote the manuscript. KN coordinated and designed the study. All authors read and approved the final manuscript. None of the authors have any competing financial or other interests in relation to this work.
